# Parental Involvement in Programs to Prevent Child Sexual Abuse: A Systematic Review of Four Decades of Research

**DOI:** 10.1177/15248380231156408

**Published:** 2023-03-17

**Authors:** Julia I. Rudolph, Sheila R. van Berkel, Melanie J. Zimmer-Gembeck, Kerryann Walsh, Drew Straker, Tia Campbell

**Affiliations:** 1 School of Applied Psychology, Griffith University, Southport, Queensland, Australia; 2 Institute for Lifecourse Development, University of Greenwich, London, UK; 3 Leiden University, Institute of Education and Child Studies, Leiden, The Netherlands; 4 Queensland University of Technology, Brisbane, Australia

**Keywords:** sexual abuse, child abuse, prevention of child abuse, family issues and mediators, treatment/intervention

## Abstract

This systematic review is the first to synthesize knowledge of parental involvement in child sexual abuse (CSA) prevention programs. Guided by the Preferred Reporting Items for Systematic Reviews and Meta-Analyses (PRISMA) criteria, 24 intervention evaluations met the inclusion criteria of aiming to change parental knowledge, attitudes, behaviors, behavioral intentions, self-efficacy, response-efficacy, or capabilities for prevention of CSA. Included papers were identified via a combination of electronic database searches (PsycINFO, Web of Science, Scopus, Google Scholar, Cochrane Library, World Health Organization’s International Clinical Trials Registry Platform, google.com.au, open.grey.eu, Global ETD, Open Access Theses & Dissertations, EThOS, and Trove) and direct communication with researchers. Improvement post intervention was found most commonly for parental behavioral intentions and response-efficacy, closely followed by parental behaviors, then capabilities, self-efficacy, knowledge, and lastly, parental attitudes. Improvements in behaviors, intentions, and response-efficacy occurred in 88 to 100% of the studies in which they were addressed, improvements in self-efficacy and capabilities occurred in 67 to 75%, and improvements in knowledge and attitudes occurred in only 50 to 56%. Many of the included evaluation studies suffered from methodological and reporting flaws, such as high participant attrition, lack of control group, lack of statistical tests, missed testing time points, and a lack of (or short) follow-up. Future parent-focused CSA prevention evaluations must address these concerns by conducting rigorous empirical research with sound methodologies and comprehensive reporting. Furthermore, study designs should consider measuring the real-world impact of increases in assessed parent variables, including their ability to prevent sexual victimization of children.

Despite many intervention programs, child sexual abuse (CSA) is experienced by many children around the world, with far-reaching personal, familial, and societal ramifications such as anxiety, depression, post-traumatic stress disorder, self-harm and suicidality, poor quality of life, loss of productivity and reduced income, alcohol and drug misuse, revictimization, family breakdown, chronic health conditions, and treatment and healthcare costs (Bonomi et al., 2009; Hailes et al., 2019; Kamiya et al., 2016; Macmillan, 2000; Stoltenborgh et al., 2011). Thus, effective prevention approaches continue to be a high public health priority. Although child-focused, school-based prevention education programs have been the most used primary prevention strategy, parental involvement has long been highlighted as an untapped resource in both academic and community spheres (Darkness to Light, n.d.; Elrod & Rubin, 1993). Yet, there has been no comprehensive systematic review of what is known about parental involvement in CSA prevention. Our aim in this review was to synthesize 40 years of parent-focused programs that aimed to improve parental CSA prevention knowledge, attitudes, or behavior as a pathway to the prevention of CSA.

## Targeting Parents with CSA Prevention Interventions

Parental involvement in child-focused CSA prevention education refers to interventions that are delivered to children but also have an adjunct parent component. It is theorized that parental participation in child-targeted interventions contributes to children’s learning gains (Kenny et al., 2008); however, parental components in school-based programs are not widely utilized. For example, a large survey by Finkelhor et al. (2014) found that, although 72% of programs in the United States included take-home materials, only 18% invited parents to be involved. Parent-focused CSA interventions, in contrast, refer to interventions specifically designed for parents, which may or may not also include a child component. Again, the use of such programs is limited. Walsh and Brandon (2012, p. 745) concluded in their review of programs in Australia, that there was a dearth of “programmatic interventions designed to support and/or encourage parents to talk with their children about sexual abuse prevention.”

There are many good reasons for including parents in intervention programs aimed to improve child safety. Parents are the most proximal members of a child’s ecology (Wurtele, 2009) and have been shown to be effective in the prevention of child maltreatment, and other child public health concerns (Altafim & Linhares, 2016; Hart et al., 2015), and are uniquely positioned to affect their child’s environment. However, evaluations of parent programs have been mixed, and methodological flaws are common. Some studies suggest that parental exposure to CSA education can result in more intended or actual parent-led sexual abuse education (PLSAE) (Binder & McNeil, 1987; Burgess & Wurtele, 1998; Kenny, 2010), while others have reported no increases in parental CSA knowledge after attending a CSA education program or at follow-up (Berrick, 1988; Briggs, 1988; Cırık et al. 2020; Reppucci et al., 1994; Rheingold et al., 2007). Evaluations of the effectiveness of PLSAE are limited, and mixed, with some studies reporting PLSAE can increase children’s knowledge and self-protection skills (Cırık et al., 2020; Jin et al., 2019; Pandia et al., 2017), but another study showing no benefit of PLSAE on child outcomes (Miltenberger & Thiesse-Duffy, 1988).

Despite calls for more parental involvement in CSA prevention, the extent of parental involvement, the type and nature of interventions targeting parents, the outcomes measured, and the effectiveness of interventions has, to our knowledge, never been systematically reviewed (see Babatsikos, 2010; Rudolph et al., 2018; Wurtele & Kenny, 2010 for narrative reviews). To understand how parent programs contribute to the CSA prevention program landscape, the purpose of this study was to review the last 40 years of research on initiatives that aimed to change parental knowledge, intentions, attitudes, self-efficacy, or behavior regarding the prevention of CSA. Specifically, there were four research questions guiding this review:

1. To what extent have parents been targeted for CSA prevention?
2. What kinds of interventions have been used to target parents?
3. What parental outcomes have been measured?
4. How effective were the interventions in achieving their objectives?

## Method

The Preferred Reporting Items for Systematic Reviews and Meta-Analyses (PRISMA) criteria (Moher et al., 2015) were used as a guideline for this systematic review.

### Search Strategy

PsycINFO, Web of Science, and Scopus electronic databases were searched in June 2021 using the following search terms: child* AND (sex* abuse OR sex* assault OR sex* violence OR sex* victim* OR rape OR molest* OR incest) AND (prevent* OR intervention OR program OR train* OR educat* OR communicat*) AND (parent* OR mother* OR father* OR caregiver* OR carer*). Google Scholar was searched with the terms child AND sex abuse OR assault AND prevent AND parent. The Cochrane Library (https://www.cochranelibrary.com) and the World Health Organization’s International Clinical Trials Registry Platform (https://www.who.int/clinical-trials-registry-platform) were searched with the abovementioned search terms. Authors with registered trials were contacted if their details were available. To identify unpublished research, google.com.au, open.grey.eu, and dissertation databases (Global ETD, Open Access Theses and Dissertations, EThOS and Trove) were searched. Finally, all first authors from included papers were contacted via email to establish whether they had access to, or were aware of, any unpublished data and/or outputs. No date limiters were applied to searches. Results were limited to papers published in English, German, and Dutch. The review was prospectively registered with PROSPERO (ID CRD42021257683).

### Inclusion and Exclusion Criteria

We included papers that reported on an intervention for the prevention of CSA. The following inclusion criteria were used:

1. Eligible participants: The prevention targets were parents or primary caregivers.
2. Eligible interventions: The intervention, program, training, or education initiative was (a) designed to change parental knowledge, attitudes, behavior, intentions, self-efficacy, response-efficacy, or capabilities regarding CSA prevention, and (b) delivered to parents in any format (i.e., face-to-face, online, mobile device application, written/visual resources, phone calls, text messages, emails, etc.).
3. Eligible outcomes: One or more of the following parental outcomes were assessed: (a) knowledge, (b) attitudes, (c) protective/prevention behaviors, (d) behavioral intentions, (e) self-efficacy, (f) response-efficacy, and (g) behavioral capabilities.

We excluded papers based on the following criteria:

1. Ineligible participants: The prevention targets were not parents or primary caregivers: (a) children were the prevention targets, (b) other adults were the prevention targets, (c) parental exposure to the intervention was incidental, or (d) parents were study informants for their children but were not directly exposed to the intervention.
2. Ineligible interventions: The intervention was not focused on CSA prevention, focusing on: (a) the prevention of other child maltreatment subtypes, (b) prevention of child maltreatment generally but not CSA specifically, (c) on prevention of other types of child victimization or violence, or (d) the intervention was not designed to change parental knowledge, attitudes, behavior, intentions, self-efficacy, response-efficacy, or capabilities regarding the prevention of CSA.
3. No intervention: Parents/caregivers were studied in the absence of a prevention intervention.
4. Ineligible outcomes: (a) Outcomes did not assess the outcomes of interest, or (b) there was no outcome measurement.

### Study Selection

**Figure 1.**
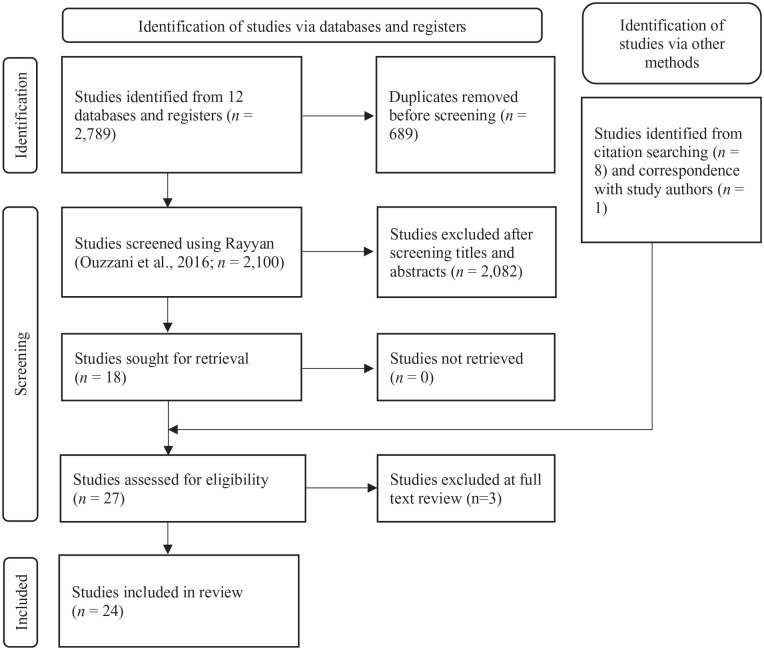
PRISMA flow diagram. *Note*. PRISMA = Preferred Reporting Items for Systematic Reviews and Meta-Analyses.

A study flow diagram is shown in Figure 1. Electronic database searches yielded 2,789 records, of which 689 were identified as duplicates. The first two authors, working independently, used Rayyan (Ouzzani et al., 2016) to double-blind screen records against the inclusion and exclusion criteria. Seventeen studies were included unanimously. There was disagreement about inclusion of three studies, which was resolved through discussion and resulted in including one additional study. Trial registers, gray literature searches, and hand-searching of included studies’ reference lists resulted in the addition of eight more studies. Email communication with corresponding authors yielded three additional studies, with one meeting the inclusion criteria. Three studies were excluded during data extraction, resulting in a final *k* = 24 included studies.

### Assessment of Study Design Quality

Independent quality assessments of the 24 studies were conducted by the first, fifth, and sixth authors, utilizing Kmet et al.’s (2004) standard quality assessment criteria. The second author checked the interrater agreement of the assessments. Quantitative studies were evaluated with responses of “yes,” “partial,” “no,” or “not applicable” to 14 statements. The statements assessed each study’s objective, design, sample, methodology, measurement outcomes, intervention evaluated, analyses, conclusions, and reporting considerations. Qualitative studies were evaluated with responses of “yes,” “partial,” and “no” to 10 statements. The statements assessed each study’s objective, design, sampling strategy, theoretical framework, methodology, analyses, verification, conclusions, and reflexivity. For both designs, responses were weighted as follows: “yes” = 2 points, “partial” = 1 point, “no” = 0 points. A final quality score was obtained by summing the response for each statement and dividing by the total possible score. Total possible scores, therefore, ranged from 0 (*lowest quality*) to 1 (*highest quality*). Statements for which the response was “not applicable” were removed, thereby reducing the total possible score for these calculations.

### Data Synthesis

Data were extracted and summarized based on the Cochrane Public Health Group (2011) *Data Extraction and Assessment Template*. In addition to methodology details, data extracted included the extant parents and nature of the parent-targeted interventions, the parental outcomes measured, and how effective these interventions have been in bringing about the desired outcomes as measured in each individual study. In this systematic review, we did not plan to conduct meta-analysis owing to heterogeneity in evaluation studies. Instead, we assembled the eligible studies, presented risk of bias assessments, and provided a detailed narrative summary to set directions for future research.

## Results

### Overview of Studies

Table 1 presents an overview of the 24 studies in chronological order, along with quality scores. Two studies were qualitative (Gesser-Edelsburg et al., 2017; Snavely, 1991) and three studies used mixed qualitative and quantitative methodologies (Berrick, 1988; Hudson, 2018, 2020). The remaining 19 studies were quantitative. Of the 22 studies with a quantitative component, 7 were randomized controlled trials (RCTs; Burgess & Wurtele, 1998; Guastaferro et al., 2020; Lak et al., 2017; McGee & Painter, 1991; Navaei et al., 2018; Nickerson et al., 2018; Rheingold et al., 2007), 5 were quasi-experimental studies (Berrick, 1988; Hébert et al., 1997, 2002; Reppucci et al., 1994; Wilkerson, 1994), and 10 used a within-group design (i.e., no comparison group; Binder & McNiel, 1987; Christian et al., 1988; Cırık et al., 2020; Hudson, 2018, 2020; Kenny, 2010; MacIntyre & Carr, 1999; Shaw et al., 2021; Smasal, 2006; Wurtele et al., 2008). Eighteen studies (75%) described evaluations of established programs (or amalgamations thereof; e.g., *ESPACE*, *Talking About Touching*, *Parenting Safe Children*, and *Second Step*). Thirteen studies (54%) were conducted in the United States, with three studies from Canada, two studies each from Iran and the United Kingdom, and one study each from Ireland, Israel, Turkey, and Zimbabwe.

**Table 1.** Papers Meeting Criteria for Inclusion (*N* = 24).

Study and Kmet Score	Method	Sample Description	Intervention	Focal Outcomes and Key Findings
Binder and McNiel (1987) Kmet score: 0.61	Within-group design; no control group; pre-test and post-test only	*n* = 60 parents (unspecified gender ratio) Age unspecified	Based on *Child Abuse Prevention Project* manual; involved workshops for children, parents, and teachers; parent workshop was 2 hours long, involved trained facilitators, and covered CSA myths and explanation of children’s workshop content	1. Parental behavior (PLSAE)—Statistically significant increases at post-test
Berrick (1988) Kmet score Quantitative: 0.46 Kmet score Qualitative: 0.40	Quasi-experiment: quantitative and qualitative; non-attending parents acted as a control group; pre-test and post-test	*n* = 116 parents (97% female) Age = 21–47 years, *M*_age_ = 33	Group education meeting facilitated by research staff teaching parents the indicators of abuse and appropriately reporting	1. Parental knowledge—Little change at post-test (statistical tests conducted but not reported) 2. Parental behavior (PLSAE)—No difference in parental behavior between the control and intervention groups at post-test, although two-thirds of the intervention group was triggered to engage in PLSAE (no statistical test) 3. Parental capability (appropriate response to suspected abuse)—No difference found at post-test (statistical test conducted but not reported)
Christian et al. (1988) Kmet score: 0.62	Within-group design; no control group; pre-test and post-test	*n* = unspecified (unspecified gender ratio) Age unspecified	SAPP: 90-minute facilitated parent group meeting (viewing the film *No More Secrets*, with discussion), plus three 30 minute sessions with children	1. Parental attitude (CSA likelihood) - No statistically significant change at post-test 2. Parental behavior (PLSAE)—Statistically significant increases at post-test 3. Parental self-efficacy (to respond to abuse and protect children)—Statistically significant increases at post-test
McGee and Painter (1991) Kmet score: 0.73	Experiment (RCT): quantitative; randomized with control group; pre-test and post-test	*n* = 376 parents (68% female) Age = 21–59 years, *M*_age_ = 33	Parents viewed either SKSK or FYFN, or neither, with or without group discussion in six intervention groups; intervention aimed to provide information about CSA, incidence, methods of intervention, etc.	1. Parental capability (appropriate disclosure response)—Statistically significant increases at post-test in appropriateness of parental actions (for all intervention groups) and support given to children (for all except SKSK groups with or without discussion) in response to hypothetical disclosures
Snavely (1991) Kmet score: 0.82	Qualitative interviews: no control group; pre-test and post-test	*n* = 13 parents (100% female) Age = 15–20 years	*Heart-to-Heart*: an 11-week group-facilitated program teaching: CSA signs; response to disclosures; and how/where to seek help	1. Parental knowledge (CSA indicators, response to disclosures, and help-seeking)—Increased at post-test (no statistical test; study qualitative) 2. Parental attitudes (responding to CSA)—Change at post-test, including increased perception of importance of communication, trust, and demonstrating affection toward children (no statistical test; study qualitative) 3. Parental behaviors—Parent behaviors were triggered at post-test, including more careful choice of babysitters, communication with community about prevention, and aiding victims of abuse to find appropriate help (no statistical test; study qualitative) 4. Parental intentions (safe behaviors, PLSAE)—Increased at post-test, including the intention to respond to disclosures appropriately, PLSAE, babysitters, and taking action to prevent abuse (no statistical test; study qualitative) 5. Parental self-efficacy (discuss CSA, respond to disclosures)—Increased at post-test; feeling more confident (no statistical test; study qualitative) 6. Parental capability (appropriate disclosure response)—Increased at post-test (no statistical test; study qualitative)
Reppucci et al. (1994) Kmet score: 0.23	Quasi-experiment: quantitative; nonrandomized control group; post-test only	*n* = 193 parents (unspecified gender) Age unspecified	CAP: no clear description; provides knowledge to parents about CSA via facilitated group meetings	1. Parental knowledge (CSA myths, CSA indicators, etc.)—No difference between the intervention group and control group at post-test (no statistical test)
Wilkerson (1994) Kmet score: 0.59	Quasi-experiment: quantitative; non-equivalent control group; pre-test & post-test	*n* = 63 parents (76.2% female) Age unspecified	*Talking About Touching for Kids and Parents*: knowledge and skills related to the prevention of neglect, physical, and sexual abuse via facilitated group meetings	1. Parental knowledge (CSA and prevention)—Statistically significant increases post-test 2. Parental attitudes (toward prevention education)—Statistically significant changes at post-test (comfort level regarding PLSAE) 3. Parental behavior—PLSAE was triggered at post-test; indicated by level of agreement between children and parents around CSA concepts being high at post-test (no statistical test)
Hébert et al. (1997) Kmet score: 0.61	Quasi-experiment: quantitative; nonrandomized control group; post-test only	*n* = 145 parents (92% female) *M*_age_ = 36.7 years	*ESPACE Parents’ Workshop*: 2-hour facilitated group workshop attempting to reduce parents’ CSA misconceptions and teaching CSA detection, and appropriate disclosure responses	1. Parental knowledge (about CSA prevalence, consequences, perpetrator characteristics, etc.)—No statistically significant difference between the intervention group and control group at post-test 2. Parental attitudes (attitudes and beliefs regarding CSA education)—Few statistically significant differences found between the intervention group and control group at post-test (only three out of 21 attitudes toward prevention concepts changed—and no attitudes from other categories)
Burgess and Wurtele (1998) Kmet score: 0.94	Experiment (RCT): quantitative; randomized with control group; post-test only (follow-up acted as post-test for variables not measured; time delay unspecified)	*n* = 45 parents (80% female) Intervention group *M*_age_ *=* 32.8 years, *SD*_age_ = 5.9; control group *M*_age_ = 32.9 years, *SD*_age_ = 8.7	*What Do I Say Now?*: a 30-minute video with actors talking to their children about sexuality and safe touching, and modeling appropriate handling of disclosure; delivered in group format with facilitated discussions	1. Parental attitude—Statistically significant increases in perception of CSA likelihood at post-test; perception of severity of CSA consequences on children did not significantly differ between groups (high base rate) 2. Parental behavior (PLSAE)—Statistically significant increases at post-test (relative to previously-measured intentions) 3. Parental intention (PLSAE)—Statistically significant increases at post-test 4. Parental self-efficacy (PLSAE)—No statistically significant difference at post-test found between the groups 5. Parental response-efficacy (PLSAE)—Statistically significant increases at post-test
MacIntyre and Carr (1999) Kmet score: 0.79	Within-group design; no control group; pre-test and post-test	*n* = 374 parents (81% female) *M*_age_ = 37 years, *SD* = 4.4	*Stay Safe Programme*: for children, parents, and teachers; parent component involved a 3-hour facilitated meeting covering myths and realities of victims and perpetrators, recognizing victims, how to help, etc.	1. Parental knowledge, attitudes and self-efficacy assessed with one measure (perpetrator and victim characteristics, CSA indicators, attitude to prevention programs, comfort/confidence responding to abuse, etc.)—Mixed results: statistically significant increases in total score on the scale at post-test, but only some individual items showed significant change 2. Parental behavior (PLSAE)—Parent–child discussion of the program reported by 90% of children at post-test (no statistical test)
Hébert et al. (2002) Kmet score: 0.77	Quasi-experiment: quantitative; nonrandomized control group; post-test only	*n* = 272 parents (87% female) Intervention group *M*_age_ = 35.8 years, *SD*_age_ = 4.0; control group *M*_age_ *=* 35.9 years, *SD*_age_ = 4.7	*ESPACE Parents’ workshop*: 2-hour facilitated group workshop addressing CSA misconceptions, teaching detection, and appropriate disclosure responses	1. Parental knowledge (perpetrator and victim characteristics, indicators, etc.)—Statistically significant increases in the intervention group compared to the control group at post-test 2. Parental intention (to report to authorities)—Statistically significant increases in the intervention group compared to the control group at post-test 3. Parental self-efficacy (to prevent)—No statistically significant difference between groups at post-test 4. Parental capability (appropriate disclosure response)—Statistically significant increases in the intervention group compared to the control group at post-test
Smasal (2006) Kmet score: 0.87	Within-group design; no control group; pre-test, post-test and 1-month follow-up (intentions only measured at post-test; behavior only measured at pre-test and follow-up, i.e., follow-up acted as post-test)	*n* = 13 parents (100% male) Age = 32 to 51 years, *M*_age_ *=* 40.6, *SD*_age_ = 6.4	Participants watched a 30-minute video, *What Do I say Now?* and took part in guided discussions and educational didactics aimed to increase PLSAE (unclear whether delivered in group or one-on-one format)	1. Parental knowledge (incidence rates, perpetrators and victims)—Statistically significant increases at post-test, which, decreased but remained higher than pre-test at 1-month follow-up 2. Parental attitudes (toward CSA prevention)—No statistically significant differences at post-test or follow-up 3. Parental behavior (PLSAE)—No statistically significant difference at post-test 4. Parental intentions (PLASE)—Statistically significant increases at post-test (relative to past actual behavior)
Rheingold et al. (2007) Kmet score: 0.92	Experiment (RCT): quantitative; randomized with control group and three intervention groups; post-test and 1-month follow-up	*n* = 200 parents (57% female) Age = 18–71 years, *M*_age_ = 32.9, *SD*_age_ = 11.0	*Stop It Now!*: featured a video and a pamphlet raising awareness of CSA, recognizing abuse, decreasing risk, and appropriate responses.	1. Parental knowledge (CSA myths, prevalence, consequences, risk factors)—Mixed results: Some statistically significant at post-test between groups and control group; but, were not maintained at follow-up 2. Parental capability (appropriate prevention methods, appropriate disclosure response, ability to recognize risky situations and CSA indicators)—Few statistically significant differences at post-test, and no statistically significant differences between the groups were maintained at follow-up (only primary prevention response appropriateness—one of six categories—increased at post-test)
Wurtele et al. (2008) Kmet score: 0.75	Within-group design; no control group; included pre-test and post-test (1 month post intervention)	*n* = 135 parents (94% female) Age = 28–56 years, *M*_age_ = 37.5, *SD*_age_ = 5.0	*Parenting Safe Children*: a 3-hour educational workshop covering CSA facts (e.g., definition, prevalence, victims and perpetrators, high-risk situations, signs and symptoms, disclosures, and environment modifications)	1. Parental knowledge (CSA myths, grooming behaviors, perpetrator characteristics, etc.)—Statistically significant increases post-test (on CSA myths and five out of six aspects of perpetrator characteristics) 2. Parental attitudes (CSA-relevant, e.g., whether CSA includes only touching)—Statistically significant change in some parental attitudes at post-test (stronger belief in children’s right to privacy and to refuse forced affection). 3. Parental behavior (PLSAE)—Statistically significant increases at post-test (on 7 out of 10 items)
Kenny (2010) Kmet score: 0.86	Within-group design; no control group; pre-test and post-test	*n* = 97 parents (94% female) Age = 24–48 years, *M*_age_ = 36.1, *SD* = 6.7	KLAS: an 8-week program of simultaneous parent and child education (utilizing Talking About Touching and What Do I Say Now?), designed for Hispanic families	1. Parental behavior (PLSAE); measured as part of a general communication and child assertiveness survey—Statistically significant increases in PLSAE at post-test for both English- and Spanish-speaking parents
Gesser-Edelsburg et al. (2017) Kmet score: 0.75	Qualitative interviews: no control group; post-test only	*n* = 20 parents (80% female) Age = 30–46 years, *M*_age_ = 39.6	*Yael Learns to Take Care of Her Body*: an edutainment play delivered to parents and children teaching tools to address CSA; identify and cope with cases of CSA.	1. Parental knowledge—Mixed results: increased CSA awareness and tools for PLSAE at post-test reported only for parents of lower SES (no statistical test; study qualitative) 2. Parental attitude (toward PLSAE)—Mixed results: parents of lower SES reported a decrease in fear about PLSAE at post-test; parents with higher SES and religious parents were more ambivalent about the play’s effectiveness (no statistical test; study qualitative)
Lak et al. (2017) Kmet score: 0.65	Experiment (RCT): quantitative; randomized into two intervention groups with no control group; pre-test and post-test (one month)	*n* = 100 mothers, (100% female) Age unspecified	Unnamed CD (Compact Disc) and group educational sessions: CD contains text, audio, images, movies, and animations; about CSA prevalence, signs. consequences and prevention	1. Parental knowledge (undefined)—Statistically significant increases for both intervention groups at post-test (education sessions only and education plus multimedia CD; no difference between groups’ increases) 2. Parental attitude (undefined)—Statistically significant increases at post-test for both intervention groups (no difference between groups’ increases)
Hudson (2018) Kmet score Quantitative: 0.55 Kmet score Qualitative: 0.55	Within-group design and qualitative; no control group; pre-test and post-test (quantitative) plus interviews with past participants (qualitative)	*n* = 252 attending as parents or professionals (79% female; 57% with at least one child) *n* = 16 past participants (qualitative; 94% female) Total sample range 16–56	*Stop It Now! Wales*: a selection of facilitated group programs aimed at engaging parents, carers, and professionals in an informed discourse about CSA and how to prevent it; the interventions were: *Parents Protect!*, *Internet Safety*, *Sexual Development in Pre and Post Pubescent Children*, *Preventing Child Sexual Abuse Exploitation*, and *Professionals Protect!*	1. Parental knowledge (CSA myths, perpetrators, victims, offending behaviors, etc.; types of knowledge measured differed by intervention group)—Increased parental knowledge at post-test (no statistical test) 2. Parental self-efficacy (to act on concerns)—Increased following interventions (no statistical test) 3. Parental response-efficacy (acting on concerns makes a difference)—Increased parental confidence that their actions would make a difference at post-test (no statistical test)
Navaei et al. (2018) Kmet score: 0.86	Experiment (RCT): quantitative; randomized with control group; included pre-test, post-test and 1-month follow-up	*n* = 62 parents (unspecified gender ratio) Age = interv-ention group *M*_age_ = 37.7, *SD*_age_ = 4.7; control group *M*_age_ = 32.2, *SD*_age_ = 3.9	Three 90-minute group counseling sessions: facilitated in accordance with GATHER consulting steps to improve parents’ self-efficacy, knowledge, attitude, and PLSAE in preventing CSA in children aged 2–6 years	1. Parental knowledge (about perpetrator and victim characteristics, probability of recurrence, etc.)—Statistically significant increases in the intervention group at post-test, retained at 1-month follow-up 2. Parental attitudes (level of agreement with the intervention)—Statistically significant in the intervention group at post-test, retained at 1-month follow-up 3. Parental behavior (PLSAE)—Statistically significant increases in the intervention group at post-test, retained at one-month follow-up 4. Parental self-efficacy (to prevent CSA)—Statistically significant increases in the intervention group at post-test, retained at one-month follow-up
Nickerson et al. (2018) Kmet score: 1.0	Experiment (RCT): quantitative; randomized with control group; included pre-test, post-test and 2-month follow-up (behavior was only measured pre and follow-up, i.e., follow-up acted as post-test)	*n* = 438 parents (87% female) Intervention group *M*_age_ *=* 39.0 years, *SD*_age_ = 6.6; control group *M*_age_ = 38.3 years, *SD*_age_ = 6.6	*Second Step*: videos, watched independently; designed to encourage parents to engage in PLSAE, and overcome barriers by providing knowledge and skills	1. Parental knowledge (CSA myths)—Mixed results: only one of three topics (restrictive abusive stereotypes) showed statistically significant increases for the intervention group at post-test, retained at two-month follow-up 2. Parental behavior (PLSAE)—A statistically significant mediated effect of the intervention on PLSAE was found at post-test—via increased knowledge predicting motivation (intentions, self-efficacy, and response-efficacy), which predicted communication 3. Parental intentions, self-efficacy, and response-efficacy (a single scale regarding PLSAE)—Statistically significant increases (measured under a single “motivation” score) in the intervention group at post-test, retained at two-month follow-up
Cırık et al. (2020) Kmet score: 0.5	Within-group design; no control group; pre-test (not for PLSAE) & post-test	*n* = 64 parents (88% female) Fathers’ *M*_age_ = 37.4 years, *SD*_age_ = 5.5; mothers’ *M*_age_ = 35.1 years, *SD*_age_ = 4.7	A training program delivered as a four-stage facilitated group workshop totaling 120 minutes, designed to teach parents about CSA and how to prevent it	1. Parental knowledge and attitudes (prevention, consequences, attitude toward prevention)—Few statistically significant at post-test (two out of 20 questions) 2. Parental behavior—PLSAE occurred following intervention (no pre-test, no statistical test)
Guastaferro et al. (2020) Kmet score: 0.96	Experiment (RCT): randomized into two intervention groups; pre-test, post-test and 1-month follow-up	*n* = 110 parents (95% female) *M*_age_ = 31 years, *SD*_age_ = 8.6	SPSHK combined with PAT: Home visiting program teaching parents about healthy child sexual development, parent–child communication about sex, and CSA-specific safety strategies	1. Parental knowledge and attitudes—Statistically significant increases in parental awareness scores (knowledge plus attitude regarding CSA prevention) for PAT+SPSHK in comparison to PAT only at post-test, maintained at 1-month follow-up 2. Parental behaviors and capabilities (protective behaviors, PLSAE, ability to identify signs of CSA)—Statistically significant increases for the PAT+SPSHK group in comparison to the PAT only group at post-test, maintained at 1-month follow-up (results suggesting adding SPHSK does not interfere with PAT efficacy)
Hudson (2020) Kmet score Quantitative: 0.22 Kmet score Qualitative: 0.46	Within-group design and qualitative; no control group; pre-test and post-test (quantitative) plus interviews within 3 months of post-test (qualitative)	*n* = 9 parents (67% female) Age unspecified	*Stop it Now! Wales*: A bespoke one-on-one intervention delivered by a parenting service for vulnerable and at-risk families, delivered weekly for a total of 6 hours	1. Parental knowledge (indicators, risk factors, etc.)—Increased at post-test (no statistical test) 2. Parental behavior (creating family safety plans)—Triggered at post-test (no statistical test) 3. Parental self-efficacy—Increased parental confidence to respond at post-test (no statistical test) 4. Parental capabilities—Increased self-reported parental capabilities at post-test to keep their children safe and skills to cope (no statistical test)
Shaw et al. (2021) Kmet score: 0.66	Within-group design; no control group; pre-test & post-test	*n* = 248 parents (90% female) Total sample—48% aged 30–40 years, 23.8% aged 40–50	*Families Matter Program*: 6 facilitated group workshops (18 hours) aiming to teach: awareness of CSA; parental role in prevention; appropriate responses to disclosures; and PLSAE	1. Parental attitudes (toward PLSAE, CSA risk, and parents’ role)—Mixed results: some statistically significant changes in parental attitudes to PLSAE at post-test (no change in CSA risk, and levels of comfort and embarrassment) 2. Parental behaviors (monitoring, PLSAE, parent-community CSA communication)—Statistically significant increases at post-test (both parent and child measures); that is, increased child monitoring, PLSAE, and conversations with people in their community about CSA 3. Parental response-efficacy (CSA prevention)—Statistically significant increases at post-test 4. Parental capability (responding to CSA and risky situations)—Statistically significant increases at post-test

*Note*. Method descriptions exclude details that do not relate to focal outcomes or parent interventions; “statistically significant” refers to *p* < .05; for some studies, focal outcomes reported are aligned with variable definitions of this review rather than the source paper. PLSAE = parent-led sexual abuse education; CSA = child sexual abuse; SAPP = *Sexual Abuse Prevention Program*; RCT = randomized controlled trial; SKSK = *Strong Kids, Safe Kids*; FYFN = *Feeling Yes, Feeling No*; CAP = Child Assault Prevention; KLAS = *Kids Learning About Safety*; SPSHK = *Smart Parents–Safe and Healthy Kids*; PAT = *Parents as Teachers.*

### Participants

Sample sizes ranged from 9 to 438 parents, with a total of over 3,400 participants. Almost all studies combined data from mothers and fathers (*k* = 17, 70%). Mothers were the sole focus of two studies (Lak et al., 2017; Snavely, 1991), one study targeted fathers exclusively (*n* = 13, Smasal, 2006) and four studies did not report participant gender. In 13 of the 17 mixed-sex studies, mothers made up more than 80% of participants; specifically, of the 3,090 parents for whom gender was reported, 528 (17%) were fathers. Two studies also included other carers, such as grandparents (Kenny, 2010; Shaw et al., 2021). One study recruited teen mothers (Snavely, 1991); and one study included only vulnerable parents (with low socioeconomic status backgrounds, sole parents, and parents having contact with statutory child protection agencies; Berrick, 1988). Ten studies (42%) reported majority White Caucasian participants, 2 reported minority-dominated samples (Kenny, 2010 had majority Hispanic participants; Snavely, 1991 had majority Black participants), and 12 studies did not report participant ethnicities.

Study participants were predominantly parents of young children. Nine studies (38%) included parents of children under 8 or 9 years (Berrick, 1988; Burgess & Wurtele, 1998; Cırık et al., 2020; Gesser-Edelsburg et al., 2017; Guastaferro et al., 2020; Kenny, 2010; Navaei et al., 2018; Wilkerson, 1994; Wurtele et al., 2008), three studies included parents of children aged 9 to 12 years (Hébert et al., 1997; Shaw et al., 2021; Smasal, 2006), two studies included parents of elementary school children (Binder & McNeil, 1987: 5–12 years; Nickerson et al., 2018: 3–11 years), and two studies included parents of children under age 18 years (Lak et al., 2017; Rheingold et al., 2007). Three studies referred to young children (Christian et al., 1988: Preschoolers; McGee & Painter, 1991: Preschoolers; MacIntyre & Carr, 1999: second and fifth graders), and five studies did not report child age (Hébert, 2002; Hudson, 2018, 2020; Reppucci et al., 1994; Snavely, 1991).

### Intervention Characteristics

Two-thirds (*k* *=* 16, 67%) of the interventions described in the included studies were designed for parents only, whereas the remaining one-third (*k* *=* 8, 33%) involved parents adjunct to child interventions. Six interventions/studies were supported by a theoretical framework: Protection Motivation Theory (Burgess & Wurtele, 1998; Nickerson et al., 2018), Bandura’s Social Cognitive Theory (Gesser-Edelsburg et al., 2017), Empowerment Model (Hébert et al., 2002), Transtheoretical Model (Smasal, 2006), and Self-Help Group Concepts (Snavely, 1991).

Interventions ranged from 1 minute (viewing a public health announcement in the *Stop It Now!* video intervention; Rheingold et al., 2007) to 18 hours (three 1-hour sessions per week for 6 weeks in the *Families Matter Program*; Shaw et al., 2021). In just over half of the studies (*k* *=* 13, 54%), interventions were delivered in school or pre-school settings (e.g., the *Yael Learns to Take Care of Her Body* play; Gesser-Edelsburg et al., 2017). Other venues were also used such as private homes (*k* *=* 2, 8%, e.g., *Parenting Safe Children*; Wurtele et al., 2008), and community centers (*k* *=* 5, 21%, e.g., the *Stop It Now!* video and pamphlet interventions; Rheingold et al., 2007). Most interventions (*k* *=* 21, 88%) aimed to increase parental knowledge about CSA, including its definition, incidence and prevalence, indicators, warning signs, risk factors, effects and consequences, victim and perpetrator characteristics, and handling of disclosures. Just over one-half of the interventions (*k* = 14, 58%) focused on PLSAE (e.g., about appropriate and inappropriate touching and safety behaviors) and how to respond to children’s questions (e.g., to answer questions honestly and age-appropriately; *k* *=* 12, 50%). Two studies (8%) also gave parents information about available community resources. One study included teenage mothers; therefore, participants were also taught how to protect themselves from CSA (*Heart-to-Heart* intervention; Snavely, 1991).

A variety of delivery modes were used, but most interventions (*k* *=* 18, 75%) involved face-to-face group psycho-educational sessions, meetings, or workshops (e.g., *Families Matter Program*; Shaw et al., 2021) including one educational play (referred to as edutainment—the *Yael Learns to Take Care of Her Body* play; Gessser-Edelsburg et al., 2017). The remaining six studies consisted of face-to-face facilitated individual sessions (two studies; e.g., *Parents as Teachers* *+* *Smart Parents—Safe and Healthy Kids*; Guastaferro et al., 2020), independent self-paced digital or online learning (two studies; e.g., the *Second Step* videos; Nickerson et al., 2018), a combination (one study; McGee & Painter, 1991), or an unspecified delivery mode (one study; Smasal, 2006).

In terms of learning strategies, four studies (17%) utilized role plays and other group interactive activities (e.g., the *Smart Parents—Safe and Healthy Kids* intervention; Guastaferro et al., 2020) and six studies (25%) supplied parents with take-home materials (e.g., *Kids Learning About Safety*; Kenny, 2010). One study utilized a multi-faceted public awareness campaign involving television and radio announcements, pamphlets, and a website (Rheingold et al., 2007). Six studies (25%; Burgess & Wurtele, 1998; Christian et al., 1988; McGee & Painter, 1991; Nickerson et al., 2018; Rheingold et al., 2007; Smasal, 2006) used educational videos teaching parents how to talk to their child about sexuality and safe touching, how to appropriately handle a CSA disclosure, and how to identify signs of CSA (e.g., *What Do I Say Now?* video; Burgess & Wurtele, 1998). Across these six studies, instructional videos ranged from 1 to 90 minutes in length. Finally, one study incorporated weekly group counseling sessions, each running for 90 minutes per week, for three consecutive weeks (Navaei et al, 2018).

### Outcomes Measured

Outcomes were assessed at pre-test and post-test (*k* *=* 14, 58%); post-test only (*k* *=* 5, 21%); post-test and follow-up (*k* *=* 1, 4%); or pre-test, post-test, and follow-up (*k* *=* 4, 17%). The shortest follow-up interval was 1 month and the longest was 2 months.

Outcomes were classified into seven categories; however, within each of the categories, there was substantial heterogeneity in the approach to measurement (see Table 1 for a description of measures). Across the 24 studies, the effectiveness of interventions was measured on a variety of parental outcomes, including knowledge, attitudes, behaviors, intentions, two forms of self-efficacy (parental self-efficacy and response-efficacy), and capabilities. Parental knowledge of CSA was defined as parents’ awareness of facts about any aspect of CSA, such as knowledge of abuse prevalence, indicators, perpetrator and victim characteristics, consequences of abuse, and prevention strategies. Knowledge was measured in 18 (75%) of the 24 studies. CSA-relevant attitudes and beliefs were defined as any parental belief, evaluation, or appraisal relevant to CSA, such as attitude toward discussing proper names for private parts with children, or attitude toward how severe the consequences of CSA are on children. Parents’ attitudes were measured in 14 studies (58%). Parental behaviors relevant to preventing or responding to CSA were defined to include parents’ own reports of behaviors they used to protect, monitor, or educate their children. Parents’ behaviors were measured in 16 studies (67%). Parental intentions to enact protective behaviors were defined as *intentions* to use monitoring, devising rules, or discussing safety. Parents’ intentions were measured in five studies (21%). Parental self-efficacy was defined as confidence in oneself to enact a behavior, and response-efficacy was defined as confidence that one’s behavior is effective or makes a difference. These two aspects of efficacy were measured in nine (38%) and four (17%) studies, respectively. Finally, parental capability was defined as skill to correctly enact a behavior and was measured in eight studies (33%).

To measure these parent outcomes before and following intervention, studies utilized numerous established (i.e., previously created by other authors) and/or custom-made self-report questionnaires (*k* *=* 21, 88%). Five studies (21%) utilized individual interviews (Berrick, 1988; Gesser-Edelsburg et al., 2017; Hudson, 2018, 2020; Snavely, 1991), three studies (15%) utilized vignette responses (Hébert et al., 2002; Rheingold et al., 2007; Wilkerson et al., 1994), and one study (Snavely, 1991) utilized role-play demonstrations in combination with participants’ personal learning journals. Twelve of the 24 studies (50%) reported on the psychometric properties of all or some of the measures used.

### Reported Results

**Figure 2.**
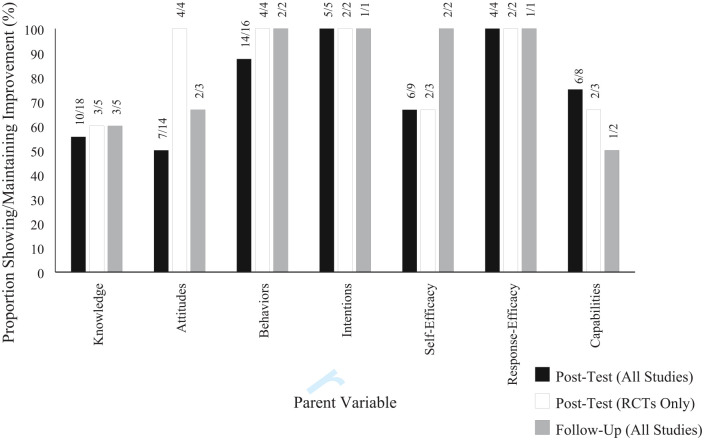
Proportion of studies reporting improvement from pre-to-post-test or reporting no change (or additional improvement) from post-test to follow-up. *Note.* Results reported as “mixed” in-text and in Table 1 were not counted as improvements; proportions were calculated as the number of studies with positive results (i.e., either finding improvement at post-test relative to either pre-test or control/comparison groups; or in the case of follow-ups, maintaining this improvement) out of the number of studies which measured the variable at the relevant time point (post-test or follow-up), where the fractions above the bars denote these numbers.

Of the 22 quantitative studies, only 15 (63%) included statistical analyses for all reported results, 3 studies (13%) lacked statistical analyses for just one variable, and 4 studies (18%) did not report statistical analyses for any result reported. Figure 2 shows the proportion of studies that reported improvement for each category of outcome, both at post-test and at follow-up. As RCTs are the gold standard for examining causal relationships, we present the results at post-test as a proportion of studies overall and a proportion of RCTs, for the purpose of comparing the findings overall with the findings of studies of high quality. This was not done for follow-up results as four of the five studies which utilized follow-up measures were RCTs. More details of these results per parent variable are explored in the following sections.

### Parental Knowledge

Parental knowledge was investigated in 18 (75%) of the reviewed studies. Of these, 10 (56%) reported improvements at post-test (three without statistical evidence, one qualitative), 4 (22%; one qualitative) reported mixed results with only some knowledge domains improved or knowledge improvements applied to only a sample subset, and 4 (22%; two without statistical evidence) reported no significant improvement in knowledge. Five studies assessed follow-up (all with statistical evidence): three of these found improvements at post-test were maintained, one found the mixed results at post-test were maintained, and one found the mixed results at post-test decreased to no-change by follow-up. The studies reporting intervention effects for knowledge used a variety of delivery methods including facilitated group sessions, audiovisual stimulus + discussion, counseling sessions, one-on-one education sessions, and home visitation. Program duration ranged from 30 minutes + discussion to 11 weeks. Some programs utilized were ESPACE, Stop It Now!, Talking About Touching, What do I Say Now?, Parenting Safe Children, and Smart Parents–Safe and Healthy Kids.

### Parental Attitudes

CSA-relevant parental attitudes were investigated in 14 (58%) of the reviewed studies. Of these studies, seven (50%) reported positive change following interventions (one qualitative), three (21%) showed mixed results (one qualitative), and four (29%) reported no improvement (all with statistical evidence). Three studies included follow-up (all including statistical evidence): two found positive results at post-test were maintained at follow-up, and one found the no-change result from post-test was maintained at follow-up. The studies reporting intervention effects for attitudes used a variety of delivery methods including facilitated group sessions, audiovisual stimulus + discussion, group workshops, and home visitation. Program duration ranged from 30 minutes + discussion to 11 weeks. Some programs utilized were Heart to Heart, Talking About Touching for Kids and Parents, and What do I Say Now?.

### Parental CSA Prevention Behaviours and Behavioral Intentions

Most studies that measured parents’ behaviors or behavioral intentions to engage in CSA prevention reported improvements following intervention. Parental PLSAE and safety behaviors were investigated in 16 (67%) of the reviewed studies. Of these studies, 14 (88%) showed increases following interventions (five without statistical evidence, one qualitative), and 2 showed no effects (one without statistical evidence). Two studies included follow-up assessment (both with statistical evidence), where both found post-test increases were maintained at follow-up. Parental intentions to enact CSA-preventative behaviors were investigated in five of the reviewed studies. All five showed increases following interventions at post-test (one without statistical evidence since evaluation was purely qualitative). Only one study included follow-up (with statistical evidence), wherein post-test increases in behavioral intentions were maintained at follow-up. The studies reporting intervention effects for behavior and intentions used a variety of delivery methods including independent learning with audiovisual stimulus, facilitated group sessions/workshops, group counseling, and home visitation. Program duration ranged from 90 minutes to 11 weeks. Some programs utilized were Stop It Now, Families Matter, Talking About Touching, What do I Say Now?, No More Secrets, and Second Step.

### Parental Self-Efficacy and Response-Efficacy

Parental self-efficacy to enact CSA-preventative behaviors was investigated in nine of the reviewed studies. Of these studies, six (67%) reported improvement following intervention (three without statistical evidence, one qualitative), one study (with statistical evidence) found mixed effects, and two found no effects (both with statistical evidence). Two studies included follow-up assessment (both with statistical evidence), wherein both found post-test increases were maintained at follow-up.

Parental response-efficacy regarding the perceived usefulness of CSA-preventative behaviors was investigated in four of the reviewed studies. Increases in parental response-efficacy were found in all four studies following interventions (one without statistical evidence). One study also assessed follow-up (with statistical evidence), which found post-test improvements were maintained at follow-up.

The studies reporting intervention effects for self- and response-efficacy used a variety of delivery methods including independent learning with audiovisual stimulus, facilitated group sessions/workshops, group counseling, one-on-one sessions, and audiovisual stimulus + group discussion. Program duration ranged from 30 minutes + discussion to 11 weeks. Some programs utilized were Stop It Now, Families Matter, What do I Say Now?, No More Secrets, and Second Step.

### Parental Capabilities

Parental capabilities to appropriately respond to CSA and disclosures or to appropriately enact protective behaviors were investigated in eight (33%) of the reviewed studies. Of these studies, six (75%) reported improvements following interventions (two without statistical evidence, one qualitative), and two found no improvement (one without statistical evidence). Two studies also included follow-up assessment (both with statistical evidence): one found post-test increases were retained, while the other reported nonsignificant improvement remained at follow-up. The studies reporting intervention effects for parental capabilities used a variety of delivery methods including viewing of audiovisual stimulus (with and without discussion), facilitated group sessions/workshops, home visitation, and one-on-one sessions. Program duration ranged from 30 minutes to 11 weeks. Some programs utilized were Feeling Yes, Feeling No, ESPACE, Smart Parents–Safe and Healthy Kids, and Stop It Now!

### Study Quality

The quality and appropriateness of the methodology and reporting for each study was evaluated against the Kmet et al. (2004) criteria (Table 1). Although there are no standardized cut-off levels for quality rankings of Kmet scores (2004), most authors define a score of >80% as “strong quality,” 70–79% as “good quality,” 50–69% as “fair quality,” and <50% as “poor quality” (Lee et al., 2020; Teixeira-Machado et al., 2019). Using these quality rankings, 6 (27%) of the 22 studies with a quantitative component included in this review were of strong quality, 5 (23%) were of good quality, 7 (32%) of fair quality, and 4 (18%) were of poor quality. The main shortcomings that were identified in the quantitative studies were lack of control groups, nonrandom allocation, and limited reporting of participant characteristics. Of the five studies with a qualitative component included in this review, one (20%) was of strong quality, one was of good quality (20%), one of fair quality (20%), and two (40%) were of poor quality. The main problems that were identified in the qualitative studies were the limited descriptions of sampling strategies, data collection methods, and data analysis methods. Other major limitations were the omissions of verification procedures to establish credibility and the reflexivity of accounts (i.e., considering the effect of the researcher’s prior experiences, assumptions and beliefs on the research process or findings).

Of the 17 studies (71%) that reported attrition (i.e., the loss of study participants over the course of the research, for example from pre-test to post-test, to follow-up), rates varied widely from 0 to 63%. The literature suggests that attrition under 5% is not likely to introduce bias, while attrition rates above 15% should be labeled as high risk of bias (Babic et al., 2019). Guided by these classifications, 5 (29%) of the studies that reported attrition fell within the low-risk category (Gesser-Edelsburg et al., 2017; Guastaferro et al., 2020; Lak et al., 2017; Navaei et al., 2018; Shaw et al., 2021), 2 (12%) studies fell within the acceptable range of 5% to 15% (MacIntyre & Carr, 1999; Wilkinson, 1994), and 10 (59%) studies fell within the high-risk category (Berrick, 1988; Burgess & Wurtele, 1998; Christian et al., 1988; Cırık et al., 2020; Kenny, 2010; Nickerson et al., 2018; Rheingold et al., 2007; Smasal, 2006; Snavely, 1991; Wurtele et al., 2008).

## Discussion

This systematic review identified 24 empirical studies investigating 18 CSA prevention interventions specifically targeting parents. A variety of intervention delivery modes was represented, with most of the programs including at least one face-to-face group session. Additionally, there was a range in intervention duration from several minutes to multiple weeks; however, most programs provided one session of around 1 to 3 hours. Although the characteristics of the programs varied substantially, all aimed to improve at least one of the following outcomes: parental knowledge, attitudes, behaviors, behavioral intentions, self-efficacy, response-efficacy, or capabilities through some form of parent education initiative.

Overall, across all seven categories of outcomes, we identified only improvement or no change in outcomes (i.e., there was no evidence of negative outcomes). These improvements in focal outcomes occurred at a minimum rate of 50% of studies which measured them (for parental attitudes), and 100% of studies at maximum (for parental behavioral intentions and response-efficacy). Other outcomes categories fell within this range (see Figure 2): knowledge (56%), self-efficacy (67%), capabilities (75%), and behaviors (88%). These results indicate that the available parent-focused CSA interventions represented in literature are effective to some extent in enhancing parent variables related to CSA prevention directly following interventions (i.e., at post-test). In terms of the longevity of the improvements, few studies (5 out of 24) included follow-up and no follow-up was longer than 2 months. However, the results showed that at one- to two-month follow-ups (where conducted), post-test results for each variable were almost always maintained (i.e., improvements remained improvements; no change remained no change).

The programs that were successful in affecting change in participant variables were heterogeneous and no similarities between them and the programs that did not yield increases in parent variables can be drawn. Effective programs included one-on-one sessions, independent study, single or multiple group sessions/workshops, and counseling. Some programs utilized audiovisual stimulus, with or without discussion, and programs ranged from 30 minutes to 11 weeks.

When comparing overall post-test results to results gained with high-quality experimental design (i.e., RCTs), the proportion of studies showing improvements was consistent for almost all variables (see Figure 2). This consistency of results overall and in comparison with RCTs exclusively suggests reliability and generalizability of the findings for those variables. One variable displayed a noteworthy discrepancy, however, with 100% of the RCTs addressing parental attitudes finding improvements. This stands in contrast to the 50% of all reviewed studies addressing parental attitudes finding improvements at post-test. These results suggest there may be an underlying factor in lower quality experimental designs that influenced results. For example, studies with no control group might leave reported attitudes among intervention groups vulnerable to undetected effects of anticipation or social desirability at pre-test, where it could be that the anticipation of receiving CSA education led to reporting of attitudes believed to be in line with those of the intervention-to-come. Otherwise, studies that lack pre-tests but instead only use control groups as reference could be vulnerable to undetected differing baseline attitudes between the control and intervention groups at pre-test, especially where randomization was not employed. This could have hidden real change otherwise observable at post-test in the higher quality research, potentially explaining the discrepancy between RCTs-only and overall results. In any case, this variability between overall results and RCT-only results suggests a degree of uncertainty.

There are some limitations in the body of research reviewed, requiring the use caution when interpreting results. Firstly, nine (38%) of the studies did not report results for all of the variables described in their method sections. Furthermore, the quality of about half of the studies was rated as low, in that of the 22 studies with a quantitative component, 7 (32%) were of fair quality and 5 (18%) were of poor quality as measured by the Kmet instrument (2004); and, of the 5 studies with a qualitative component, 1 was assessed to be of fair quality (20%) and 2 (40%) were of poor quality. Ten studies (42%) were also considered at high-risk for bias due to high attrition rates (up to 63%), with 7 studies not reporting attrition rates. Many of the included studies also suffered from methodological flaws, such as the lack of clear theoretical frameworks, the absence of control groups, absence of pre-tests and/or follow-ups, and limited follow-up timeframes.

There was a general lack of adequate reporting in the reviewed studies and future research should provide more detailed information concerning the program being investigated, the theoretical underpinnings of interventions, participants, study design, and the theory and mechanisms of change (congruent with guidelines such as the PRISMA extension statement and TIDieR checklist for intervention description). Furthermore, this review demonstrates that future research should aim to use control groups, include three points of data collection (pre-tests, post-tests, and follow-ups), use follow-ups at timespans beyond 2 months, and employ methods to prevent attrition.

Of further concern is the issue of diversity, with 66% of the included studies originating from North America and 71% representing English-speaking populations. Likewise, fathers were grossly underrepresented, making up only 17% of participants included in this review. Future research should prioritize recruiting fathers where possible (as was done by Smasal, 2006), perhaps by recruiting from male-dominated social spaces; however, it is understood that cultural factors impinge on this somewhat. None of the studies included in this review reported participants’ sexual orientation or gender identification, and future research into parental involvement in CSA prevention would benefit from attempting to understand the unique contributions and challenges faced by parents in the LGBTIQA+ community. This could be achieved by involving LGBTIQA+ support organizations in recruitment campaigns.

Finally, only two of the included studies in this review (Cırık et al., 2020—no statistical analyses; Wilkerson, 1994—unpublished dissertation) assessed the downstream effect of a change in parent variables on child outcomes. For example, an important measure of effectiveness is confirming whether the reported increases in PLSAE following parental program attendance, resulted in enhanced child knowledge of sexual abuse prevention strategies. Furthermore, evidence of increases in parental variables (knowledge, attitudes, behavior, etc.) does not necessarily equate to the prevention/reduction of CSA. Without empirical evidence or grounded theory on which parental factors may protect children from sexual abuse, effectiveness of parent-focused CSA prevention programs cannot ultimately be assessed. With a view to investing scant public resources in the best possible initiatives, future research should at least consider whether the parent variables being measured are genuinely influential in preventing CSA and improving parental responses to it. For example, Rudolph and colleagues suggest that parents may be better employed as protectors rather than educators, proposing that parents can be protective via the creation of safer environments and the enhancement of child well-being (Two Pathways Model; Rudolph et al., 2018; Rudolph & Zimmer-Gembeck, 2018).

As the wider costs and/or benefits of parent education to encourage PLSAE have not been measured, we can only speculate; it is possible that PLSAE has wider benefits for children such as increasing parent–child communication about sensitive topics in general, enhancing parent–child relationships and heightening the likelihood that a child will disclose unwanted or abusive encounters. However, conversely, it is also possible that PLSAE results in unintended side-effects such as worry, fear, anxiety, loss of trust, wariness of touch, and wariness of familiar and unfamiliar adults (Rudolph, 2022).

In summary, this review suggests that parent-focused CSA prevention programs are generally effective in facilitating change in measured parent variables such as knowledge, attitudes, and behaviors. However, some of these findings were borne of research suffering from methodological flaws, so caution should be used in their interpretation. More research is necessary to draw firmer conclusions on the efficacy of such programs on parent variables, as well as to detect the extent to which changes in assessed parent variables affect the desired change in child outcomes, and ultimately, whether enhancing these parent variables has real-world preventive value.

**Table:** 

*Critical Findings*
• 20 published and 4 unpublished studies conducted since 1980 were reviewed. • The majority of studies were Anglo-centric, with two-thirds conducted in North America and almost three-quarters conducted in English-speaking countries. • Fathers were underrepresented in evaluations, making up 1 out of every 6 participants. • Improvement post intervention was found most commonly for parental behavioral intentions and response-efficacy, closely followed by parental behaviors, then capabilities, self-efficacy, knowledge, and lastly, parental attitudes. • Most program evaluations of parent-focused programs had serious methodological limitations.
*Implications for Policy, Practice, and Research*
• Evaluation research demonstrates that programs have the capacity to change parental knowledge, attitudes, behaviors, behavioral intentions, self-efficacy, response-efficacy, and capabilities for prevention of CSA. • Future research must address the methodological limitations that hamper conclusions being drawn for a large proportion of available research. • High levels of attrition in included studies suggest that more could be done to engage parents and prevent drop out. • Attempts should be made to give populations neglected in previous research a voice. • The real-world impact of parent-focused CSA education should be considered.
